# The Pleiotropic Effects of the GM-CSF Rheostat on Myeloid Cell Differentiation and Function: More Than a Numbers Game

**DOI:** 10.3389/fimmu.2019.02679

**Published:** 2019-11-15

**Authors:** Yifan Zhan, Andrew M. Lew, Michael Chopin

**Affiliations:** ^1^The Walter and Eliza Hall Institute of Medical Research, Parkville, VIC, Australia; ^2^Department of Medical Biology, University of Melbourne, Parkville, VIC, Australia; ^3^Department of Immunology and Microbiology, University of Melbourne, Parkville, VIC, Australia

**Keywords:** GM-CSF, macrophages, dendritic cells, differentiation, function

## Abstract

Granulocyte Macrophage-Colony Stimulating Factor (GM-CSF) is a myelopoietic growth factor that has pleiotropic effects not only in promoting the differentiation of immature precursors into polymorphonuclear neutrophils (PMNs), monocytes/macrophages (MØs) and dendritic cells (DCs), but also in controlling the function of fully mature myeloid cells. This broad spectrum of GM-CSF action may elicit paradoxical outcomes—both immunostimulation and immunosuppression—in infection, inflammation, and cancer. The complexity of GM-CSF action remains to be fully unraveled. Several aspects of GM-CSF action could contribute to its diverse biological consequences. Firstly, GM-CSF as a single cytokine affects development of most myeloid cells from progenitors to mature immune cells. Secondly, GM-CSF activates JAK2/STAT5 and also activate multiple signaling modules and transcriptional factors that direct different biological processes. Thirdly, GM-CSF can be produced by different cell types including tumor cells in response to different environmental cues; thus, GM-CSF quantity can vary greatly under different pathophysiological settings. Finally, GM-CSF signaling is also fine-tuned by other less defined feedback mechanisms. In this review, we will discuss the role of GM-CSF in orchestrating the differentiation, survival, and proliferation during the generation of multiple lineages of myeloid cells (PMNs, MØs, and DCs). We will also discuss the role of GM-CSF in regulating the function of DCs and the functional polarization of MØs. We highlight how the dose of GM-CSF and corresponding signal strength acts as a rheostat to fine-tune cell fate, and thus the way GM-CSF may best be targeted for immuno-intervention in infection, inflammation and cancer.

## Introduction

The Granulocyte Macrophage-Colony Stimulating Factor (GM-CSF) is a small glycoprotein that is able to stimulate generation of polymorphonuclear neutrophils (PMNs) as well as mononuclear monocytes, macrophages (MØs) and dendritic cells (DC) (1–3). When added to mouse bone marrow precursors *in vitro*, GM-CSF acts in two phases: an early differentiating phase of PMNs and CD11b^+^ mononuclear cells from progenitors, and a late phase of MØs and monocyte-derived DCs (moDC) from CD11b^+^ mononuclear cells. For several decades it has been known that the outcome of such cultures is greatly influenced by a number of factors, including cell density, the presence of stromal cells, co-stimulatory signals, the serum quality and the concentration of GM-CSF (1). Despite this, the molecular mechanisms underpinning the heterogeneity of the myeloid cells produced in these GM-CSF induced cultures are still ill defined. For example, while cytokines such as IL-4, IL-13, TNF-α, TLR ligands or even GM-CSF concentration could alter dramatically the ratio of generated myeloid cells (1, 4–6), the nature of this bias under different conditions has not been fully resolved at a molecular level. It has also not been fully resolved whether differentiation fate under these conditions is the result of either plasticity between MØ and moDC, or the selective expansion of a committed precursor under favorable conditions of culture. While GM-CSF is extensively used in supporting myelopoesis *in vitro*, the role of GM-CSF *in vivo* remains obscure. GM-CSF deficiency has little impact on myeloid cells except for the impairment of alveolar MØs *in vivo* (7–10). Nevertheless, in transgenic mice harboring high levels of GM-CSF (GM-CSF-Tg), myelopoiesis is substantially increased (11, 12).

While the importance of GM-CSF for myelopoiesis *in vivo* remains a matter of debate, there is cogent evidence that GM-CSF is an important mediator in inflammatory conditions such as during infection and tumor immunity (13–16). These studies suggest a role for GM-CSF in regulating biological functions of fully mature cells. Studies on GM-CSF have mainly focused on its pro-inflammatory role. Nevertheless, GM-CSF has also been linked to immuno-suppression, particularly in tumor setting. Thus, exposure of myeloid cells to GM-CSF can lead to sharp opposite extremes, and these contrasting effects of GM-CSF on myeloid cells remains hitherto unexplained.

The GM-CSF receptor (GM-CSFR) is composed of a ligand-specific alpha chain and a beta chain common with IL-3 and IL-5. Despite sharing this signaling beta chain, IL-3 or IL-5 engagement leads to distinct signaling events and myeloid cell outcomes (17). For example, IL-3 is mostly associated with differentiation of mast cells/basophils, while IL-5 is associated with differentiation of eosinophils (17). GM-CSFR is found on most myeloid cells including their precursors. Upon engagement, GM-CSFR elicits JAK2 phosphorylation, which triggers multiple intracellular signaling pathways, including STAT5, PI3K, and MAPK (15, 18). Of note, GM-CSF can selectively turn on signaling modules in a dose-dependent fashion, and can therefore differentially impact cell survival, proliferation, and differentiation at different doses (15, 18–20). GM-CSF has been shown to activate and/or upregulate many transcriptional factors such as the STAT proteins, PU.1 and interferon regulatory factors (IRFs) (18). Such factors have been implicated in the differentiation and function fate determination of myeloid cells, but it is not clear how induction and function of these transcription factors are linked to GM-CSF signaling strength.

Apart from GM-CSF abundance, GM-CSF signaling strength can be influenced by multiple factors, including post-translational modification. For example, glycosylated GM-CSF has less immunogenicity and greater *in vivo* pharmacokinetic availability than its non-glycosylated form Gribben et al. (21). Nevertheless, glycosylation of GM-CSF is not required for its biologic activity *in vitro* (22). In contrast, the GM-CSF receptor α subunit requires N-glycosylation for binding and signaling (23, 24). Thus, it has been speculated that glycosylation of the α subunit may modulate cellular responsiveness to GM-CSF (24). In addition, GM-CSF receptor signaling can also be regulated by the suppressors of cytokine signaling proteins (SOCS family members). However, the consequences of SOCS signaling in controlling GM-CSFR signaling strength and therefore myeloid cell differentiation and/or function have been little explored.

**Figure 1:**
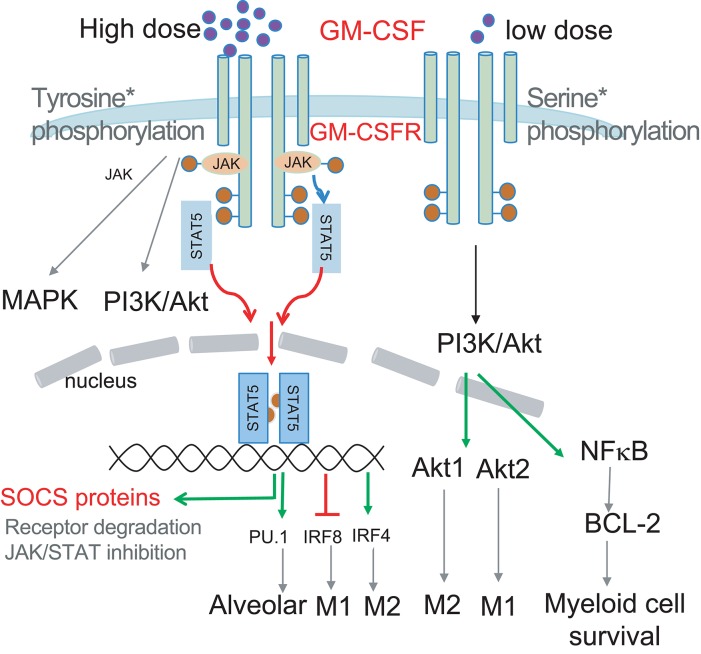
Schematic illustrating how GM-CSF dose selects the signal modules to be activated. Low dose GM-CSF signaling triggers Ser phosphorylation of the GM-CSFR beta chain, PI3K/Akt activation and subsequent BCL-2 enhanced survival. High dose signaling triggers Tyr phosphorylation of the GM-CSFR beta chain resulting in JAK/STAT5 activation, leading to upregulation of transcription factors PU.1 and IRF4, and downregulation of IRF8 to impact differentially on myeloid cell differentiation and function. JAK2/STAT5 activation by GM-CSF could also induce transcription of SOCS proteins to negatively regulate signaling to form a signaling regulatory loop. PI3K activation can also contribute to MØ polarization via preferential activation of Akt1 and Akt2.

In this review, we will highlight the dynamic changes in GM-CSF quantity in different pathological situations and dose-dependent differences in the biological response to GM-CSF, ranging from immunostimulating to immunosuppressive. We dissect the differential impact of GM-CSF on the main types of myeloid cells. As the upstream events of GM-CSF signaling and the inflammatory biological outcomes have been reviewed elsewhere (17, 20), we will highlight the potential role for negative feedback regulators on GM-CSF signal strength and downstream transcriptional factors that influence myeloid differentiation trajectory and function (Figure 1). Furthermore, we will discuss the contribution of PI3K and downstream NFκB pathways upon GM-CSF engagement in controlling not only myeloid cell survival (19) but also macrophage polarization via the differential involvement of Akt1 and Akt2 subunits (25). Finally, we also discuss the role of GM-CSF in regulating end-cell function, particularly functional polarization of MØs. In the process, we aim to shed some light on the paradoxical role of GM-CSF in immune regulation and facilitate the agonistic and antagonistic targeting of GM-CSF as an immuno-intervention in infection, inflammation, and cancer. As this review covers mouse and human studies, we have indicated the species when human GM-CSF is discussed.

## Dynamic Production of GM-CSF: How Much Is Produced *in vivo*?

The amount of GM-CSF is likely to be a key factor in determining its biological activity (19, 26). Thus, we will briefly describe the main sources of GM-CSF. A diverse set of hematopoietic and non-hematopoietic cells have been shown to secrete GM-CSF. They include T cells (27–30), human natural killer cells (31), mast cells (32), monocytes/MØs (33), human endothelial cells (34), and human fibroblasts (35). The relative contribution of each individual subset to the overall GM-CSF produced under steady-state or inflammatory conditions has not been determined. In the lung, production of GM-CSF by endothelial cells in the steady state was instrumental in the differentiation of alveolar MØs from fetal monocytes (10, 36). Under inflammatory conditions, such as collagen induced arthritis and experimental autoimmune encephalomyelitis (EAE), the production of GM-CSF by T cells has been reported to promote disease progression (28–30), although there is contention about the role of GM-CSF in EAE pathology (37). On the other hand, GM-CSF derived from radio-resistant wild-type cells in GM-CSF^−/−^ bone marrow reconstituted irradiation chimera was sufficient to confer resistance to infection with *Mycobacterium tuberculosis* (38). GM-CSF is often used in the range of 10–20 ng/ml for *in vitro* myeloid cell differentiation (2–4, 39). It raises the question—what levels of GM-CSF can be reached *in vivo*? In physiological situations, concentrations of around 20 pg/mL of GM-CSF could be detected in human serum (40). Under pathological conditions, human GM-CSF was found to be significantly elevated in the serum and tissues in inflammatory diseases such as rheumatoid arthritis and colitis (41–43). GM-CSF increase was also observed in mice following LPS administration (44) and during bacterial infection (45). Notably, GM-CSF quantity can reach and persist at >10 ng per lung of mice infected with *M. tuberculosis* (38). When human GM-CSF was used for myeloid recovery after chemotherapy and bone marrow transplantation, patients were given with >32 μg/kg body per day for 14 days (46).

## Solid Tumor Derived GM-CSF: Common Feature?

It has long been appreciated that tumor cells can produce a variety of cytokines and chemokines (47). The Broad Institute cancer cell line encyclopedia database (https://portals.broadinstitute.org/ccle) shows that a broad spectrum of solid tumor cell lines express human GM-CSF mRNA. For example, tumor cells from the kidney, pancreas and gastrointestinal tract displayed prominent GM-CSF transcription. Concordantly, an early study showed that about a third of the 75 human tumor lines tested secreted GM-CSF; this comprised a large proportion of lines from renal, prostate and colon cancers and a modest proportion of breast, cervical, ovarian and melanoma cancers (47). Indeed, 10^5^ W-RCC renal cancer cells produced a remarkable 39 ng/mL after 16 h in culture (47). A mouse renal tumor line RenCa also produced about 0.5 ng GM-CSF/10^6^ cells/24 h (48). In another study, a panel of mouse pancreatic ductal adenocarcinoma (PDA) tumor cell lines all produced GM-CSF (60–500 pg/mL) while benign pancreatic ductal cells did not (49). These results indicate that GM-CSF production by human and mouse tumor cells may not be uncommon.

## Role of GM-CSF in Tumor, Autoimmunity/Inflammation and Infection: Stimulating or Suppressive?

Several reviews have described that GM-CSF has a profound immune regulatory role in health and disease (13–17). Here we briefly discuss the role of GM-CSF in tumor, autoimmunity/inflammation, and infection, with the aim to contrast the opposite roles of GM-CSF in immune regulation.

### GM-CSF Promotes and Suppresses Tumor Immunity

The use of murine tumor cells genetically modified to secrete cytokines has established GM-CSF as a strong immune adjuvant for vaccination to promote anti-tumor immunity (50). In a vaccination setting, Zarei et al. showed that tumor derived GM-CSF was sufficient to recruit DCs to the vaccination site in murine tumor models, thereby promoting a strong anti-tumor response and protecting from further tumor challenge (48). Hence, clinical trials using human GM-CSF as an immune adjuvant in cancer patients have been conducted with some promising outcomes (51–53). However, the use of human GM-CSF at high doses may lead to advert events such as immunosuppression (54). In mouse models, tumor derived GM-CSF has also been shown to promote the development of myeloid derived suppressor cells (49). Consequently, neutralization of GM-CSF has also been shown to reduce suppressive cells and limit tumor growth (49). Furthermore, tumor derived GM-CSF can also act in an autocrine manner to sustain tumor growth (55). Thus, GM-CSF secretion within the cancerous tissue may have very contrasting effects on either promoting anti-tumor immunity, suppressing anti-tumor immunity or promoting tumor growth directly. It is likely that the temporal and spatial abundance of GM-CSF, together with the machinery controlling GM-CSF signal strength including receptor expression and regulatory circuitry would dictate the cellular and biological outcome of tumor derived GM-CSF.

### GM-CSF Promotes and Suppresses Autoimmunity

Evidence that GM-CSF is pro-inflammatory in several autoimmune diseases comes from various studies: (1) treatment with human GM-CSF to correct neutropenia results in flare-ups of rheumatoid arthritis (56, 57); (2) human GM-CSF was present in lesions of rheumatoid arthritis (41); and the cerebrospinal fluid of MS patients (58); (3) GM-CSF deficiency confers resistance to experimental collagen induced arthritis (59) and EAE (60) in mouse models. In line with the above studies, anti-GM-CSF mAb treatment was found to be effective at ameliorating the ensuing disease in mouse models, partly by reducing myeloid cell infiltration (61, 62). In clinical trials, anti-human GM-CSF mAb namilumab and MOR103 demonstrated evidence of efficacy in active rheumatoid arthritis (63, 64). Similarly, human trials of anti-GM-CSF receptor α mAb Mavrilimumab on rheumatoid arthritis had also been shown to reduce disease activity (65, 66).

However, GM-CSF is not always detrimental in autoimmune settings and has also been shown to be beneficial via the suppression of undesired immune responses (67). The supporting evidence includes: (1) treatment with human GM-CSF ameliorates Crohn's disease (68); (2) GM-CSF prevents diabetes development in NOD mice by promoting immature tolerogenic DCs and controlling the number of regulatory T cells (69); (3) GM-CSF deficiency in mouse results in the development of lupus-like disorder (70) while combined deficiency of GM-CSF and IL-3 results in the development of autoimmune diabetes (71). The cellular and molecular basis for these beneficial effects of GM-CSF is not clear. As discussed in a recent review (67), there are at least two potential mechanisms for GM-CSF to suppress autoimmunity. Firstly, GM-CSF can induce DCs and macrophages to activate antigen-specific Tregs and suppresses experimental autoimmune disease in autoimmune thyroiditis (72). GM-CSF-autoantigen conjugates had been found to be particularly effective to expand Tregs in an EAE model (73). GM-CSF can even directly expand *in vitro* induced Tregs to suppress disease development in a cell transfer model of type 1 diabetes (74). Secondly, GM-CSF can induce the production of monocytes with suppressive functions that dampen disease induction and severity in an IRF1 dependent fashion (75). Beyond autoimmunity, MØs can also be detrimental or beneficial to graft tolerance in organ transplantation (76). In such a context, it is interesting to note that GM-CSF mediates graft-vs. -host disease but not graft-vs. -leukemia responses, suggesting an intervention opportunity targeting GM-CSF in allogenic hematopoietic cell transplantation (77).

### GM-CSF Promotes Immunity and Mediates Immunopathology During Infection

Studies in mice deficient in GM-CSF and GM-CSFR have highlighted the critical role for GM-CSF and its receptor in maintaining alveolar MØs in the lung (7, 10, 36, 78). Many studies have established that GM-CSF has a non-redundant role in promoting anti-pathogen immunity. Deficiency in GM-CSF reduced emergency myelopoiesis and reduced *Listeria* and *M. tuberculosis* protection in mice (79, 80). Concordantly, GM-CSF treatment enhanced protective immunity against infection with *M. tuberculosis* and *Salmonella typhimurium* (81, 82). GM-CSF also promoted resistance against various parasite infections including blood-stage malaria (83), trypanosomiasis (84), and leishmaniasis (85). Interestingly, the combined blockade of GM-CSF and IL-3 prevented the development of cerebral malaria (86). Notably, infection in human and mouse models can also lead to immunosuppression (87–91). Unfortunately, although these studies indicated an association with the generation of immunosuppressive myeloid cells, full understanding on how GM-CSF shapes immunosuppression remains elusive.

## Dose-Dependent Differentiation of PMNs and MØs?

The exposure of bone marrow progenitors to GM-CSF leads to the production of two functionally distinct myeloid cells: PMNs and MØs. What determines the deviation to PMN vs. MØ pathway? In early studies using *in vitro* agarose cultures, high GM-CSF concentrations favored PMNs differentiation, whereas low concentrations favored MØ differentiation (26, 92); this effect was termed “differentiation downgrading.” Interestingly, a recent article has provided a mathematical interpretation for this observation, enabling the reproduction of the concentration dependent pattern of GM-CSF induced differentiation based on induction of key transcriptional factors controlling lineage commitment (93). However, when GM-CSF signaling strength that is represented by both GM-CSF quantity and receptor density is high over time, monopoiesis is favored over granulopoiesis (93). In line with this predictive model, our recent data showed that high dose GM-CSF favored monopoiesis over granulopoiesis *in vitro* (5). Similarly, GM-CSF transgenic mice had preferential expansion of MØs in multiple organs (5, 11, 12). Consistent with the findings above, van Nieuwenhuijze et al. described increased MØs compared to PMNs in transgenic mice expressing high level of GM-CSF (12). Conceivably, GM-CSF signal strength is not only reflected by the ratio GM-CSFR:GM-CSF but also by intracellular mechanism controlling GM-CSF signaling. We contend that all these factors ultimately play a critical role in determining myeloid cell differentiation.

## Differential Requirement of GM-CSF for Survival of MØs and PMNs?

Human PMNs rapidly lose viability in culture (94). Human GM-CSF but not G-CSF, IL-6, and IL-8 prevented apoptosis of PMNs, prolonging *in vitro* survival (94). Of note, despite sharing the βc receptor with GM-CSF, IL-3 did not improve cell survival, likely due to low expression of IL-3 receptor on mature PMNs (94, 95). We observed that the addition of small quantities of GM-CSF *in vitro* (80 pg/mL) can lead to substantially increased survival of murine blood PMNs (5). Interestingly, a detailed analysis of the signaling pathway induced by such low levels of GM-CSF have shown that it was sufficient to activate Ser585 of the GM-CSFR, thereby promoting downstream signaling events, in particular the PI3K-Akt pathway, that led to increased cell survival (19, 96). As pro-survival members of the BCL-2 family including BCL-2, BCL-xL, A1, MCL-1, and BCL-w have a key role in maintaining the viability of most immune cells (97), the precise contribution of individual molecules to PMN survival, specifically GM-CSF enhanced PMN survival, is unclear. Human GM-CSF has been shown to increase expression of BCL-2 but not BCL-xL in one study (96) while it increased BCL-xL transcription in another study (98). Functionally, antagonism of BCL-2 or BCL-xL has had some effects on mouse and human neutrophil count *in vivo* (99, 100). Similarly, A1, identified as a GM-CSF induced molecule (101), showed a pro-survival role for PMN in some studies (5, 102) but not in the most definitive study where all the functional A1 genes were ablated (103). In addition, human GM-CSF could promote granulocyte survival by maintaining MCL-1 stability (104). It is somewhat puzzling that human GM-CSF can also induce the expression of the pro-apoptotic BCL-2 family member Bim in human and mouse PMNs via a PI3K dependent fashion (105). Compared to PMNs, monocytes/MØs had better spontaneous survival in culture, and survival enhancement by GM-CSF was less remarkable than the effect observed on PMNs (5). The loss of either MCL-1 or A1 has a limited effect on murine monocyte/ MØ survival (103, 106). Overall, GM-CSF has a prominent role in promoting survival of myeloid cells. However, the molecular events responsible for the differential survival properties observed for PMNs and monocytes/MØs, with or without GM-CSF remain ill explained. Furthermore, there is little known about the role of GM-CSF in regulating multiple non-BCL-2 regulated cell death pathways including death-receptor regulated apoptosis, necroptosis and autophagy.

## Differential Impact of GM-CSF on Differentiation of MØs and moDCs: Plasticity or Selective Expansion?

GM-CSF is routinely used to generate large numbers of dendritic cells from mouse bone marrow or human monocyte cultures (2, 3, 107). Yet recently, CD11c^+^ mononuclear cells generated in the former culture were found to contain two main populations: CD11c^+^MHCII^int^CD11b^hi^ CD115^hi^Flt3^−^ MØs and a MHCII^hi^CD11b^int^ cell fraction enriched for Ftl3^+^ DCs (4). MØs and DCs within CD11c^+^ mononuclear cells not only differ in their gene signature but also function (4). MØs have a high capacity for producing proinflammatory cytokines while DCs have a high capacity for presenting antigens (4). In addition, recent evidence highlighted that the inflammasome activity of such cultures was due to MØs, not DCs (108).

Ontogeny analyses elegantly showed that macrophage-dendritic precursors, common monocyte progenitors, common dendritic cell progenitors, and Ly6C^high^ monocytes can all become MØs or DCs, with different expansion and differentiation rates (4). Of note, Flt3^+^CD11c^−^ MHCII^+^ PU.1^hi^ cells within the Ly6C^+^ monocyte subset have been identified as precursors of GM-CSF dependent moDCs (109). Notwithstanding, there are still many unanswered questions regarding the conditions determining the differentiation fate of MØs and DCs.

### GM-CSF Signaling Strength

GM-CSF signal strength is the net result of GM-CSF quantity, GM-CSFR expression level and positive/negative regulatory circuitry controlling GM-CSF signaling. Most *in vitro* studies use a range of 5–20 ng/mL GM-CSF to drive DC differentiation, with variation in cell density and culture duration. It had been shown that low dose of GM-CSF promotes the development of immature DCs featuring tolerogenic function (110). Using the recent definition of MØs and DCs within CD11c^+^ cells generated in GM-CSF culture (4), we and others noticed that an intermediate dose of GM-CSF favored moDC differentiation while higher doses of GM-CSF favored macrophage differentiation (5, 111). As alluded to earlier, the GM-CSFR could work as a binary switch: low doses of GM-CSF led to Ser phosphorylation, whereas high doses led to Tyr phosphorylation and STAT5 activation (19). However, it remains unclear on how this binary switch contributes to DC and MØ differentiation.

In addition to the interpretation of the abundance of the ligand, the GM-CSF induced signaling cascade can be regulated by negative regulators of cytokine signaling. One such example is the degradation of GM-CSFR through SOCS1 mediated by ubiquitination (112). Yet, the consequences of SOCS1-mediated GM-CSFR downregulation has not been examined in the context of DC differentiation. In response to GM-CSF, myeloid cells are induced to express another member of the SOCS family, CISH (113–115). CISH knockdown by shRNA was shown to impede GM-CSF-induced DC development and DC function (115). However, as authors demonstrated that CISH knockdown suppressed precursor cell proliferation, it is still unclear if CISH knockdown can directly impact on the differentiation of MØs and DCs.

Taken together, we speculate that GM-CSF induced signaling strength dictates cellular outcome, with moderate GM-CSF signaling strength enabling DC differentiation while strong GM-CSF signaling strength favors MØ differentiation.

### Promotion of DC Differentiation by IL-4 and Other Stimuli: Fate Plasticity?

Even at the monocyte stage when cell proliferation is very limited (4), human and mouse GM-CSF, particularly with IL-4, can differentiate human and mouse monocytes into DCs (4, 107, 109). It raises the question of whether IL-4 alters the differentiation fate for cells destined to become MØs in its absence, implying a certain degree of fate plasticity within that compartment. Consistent with the idea of a certain degree of plasticity, IL-4, through the activation of the transcription factor STAT6, has recently been shown to induce demethylation of genes favoring DC differentiation and enforced STAT6 activation in the absence of IL-4 also favors DC differentiation (116). Interestingly, the transcription factor PU.1 has been shown to be required for the induction of STAT6-mediated transcription (117) and to promote DC generation from monocytes while inhibiting MØ production (109). Thus, PU.1 and STAT6 could abet terminal DC development. However, individual STAT proteins seldom act in isolation such that functional balance between multiple STAT proteins is important to determine cell differentiation (118). Interestingly, the effects of IL-4 on GM-CSF induced DC differentiation was shown to be dependent on the dose of both IL-4 and GM-CSF (119), suggesting that differentiation trajectories are dependent on the signal strength of both cytokines. Of note, IL-4 not only altered the differentiation trajectory under GM-CSF but also increased APC function of generated dendritic cells (120). IL-4 induced the expression of IRF4 that was not only critically required for DC differentiation, but also for their antigen cross-presentation capacity and the expression of costimulatory molecules (120).

An IL-4 related Th2 cytokine IL-13 has also been shown to enhance GM-CSF stimulated DC differentiation from mouse bone marrow cells (119) and human monocytes (121, 122), although the potency and action of IL-4 and IL-13 may differ. Furthermore, TNF-α and LPS added at a late stage of bone marrow cell culture with GM-CSF have also been shown to promote DC differentiation/maturation (2, 3). At least for TNF, multiple STAT proteins including STAT6 can be activated upon stimulation. Overall, there is considerable plasticity for GM-CSF induced differentiation of mononuclear cells, subject to the conditions that activate signaling modules favoring either DC or MØ differentiation.

### Importance of GM-CSF for *in vivo* moDC Differentiation

Despite the strong potency of GM-CSF to induce DC differentiation *in vitro*, GM-CSF and its receptor are redundant for the differentiation of moDCs *in vivo*, at least during acute infection and inflammation (9, 123, 124). It could be that infection and inflammation induce high levels of many cytokines including M-CSF and TNF-α that could influence moDC differentiation and therefore mask the role of GM-CSF. In situations where GM-CSF concentration increase is more selective (e.g., GM-CSF overexpression or engraftment of a GM-CSF-producing tumor) (109, 125), GM-CSF seems to have a positive role in inducing moDC differentiation. In an EAE model with Th17 transfer, GM-CSFR^−/−^ moDC infiltrates in CNS tissue were significantly reduced in a competitive scenario (126). Our view is that GM-CSF is sufficient but not essential for production of moDCs *in vivo*. Its importance on moDCs *in vivo* may instead be more critical for their effector function (see below).

### Impact of GM-CSF on Non-moDCs

Many decades of work have established that the dendritic cell network is heterogenous and consists of many subsets with different phenotypic and functional features (127–129). DCs, excluding moDCs, have recently been categorized into three groups: cDC1s (for both CD8^+^ and CD103^+^ DCs), cDC2s (for CD11b^+^ and CD172α^+^), and pDCs (130). Despite the differentiation of these cells being largely independent of GM-CSF, GM-CSF has pleiotropic impacts on all these DC subsets. In Flt3L-supplemented cultures of bone marrow cells, inclusion of low dose GM-CSF (0.3 ng/mL) increased the production of cDC1s, cDC2s, and pDCs, while neutralization of endogenous GM-CSF reduced all DC generation (131). Similar findings have also been derived *in vivo*, particularly in mice with combined loss of GM-CSF and Flt3L (132). Enhancement of overall DC differentiation by GM-CSF is likely due to the positive effect of GM-CSF on progenitor commitment to myeloid lineages and expansion of such progenitors. However, at high doses of GM-CSF, development of cDC1s and pDCs under Flt3L stimulation was severely hampered (133, 134). At least for pDCs, it was shown that strong GM-CSF signaling leads to strong STAT5 activation and suppression of IRF8 transcription, which is critical for pDC differentiation (134). cDC1s include both lymphoid CD8^+^ DCs and tissue CD103^+^CD207^+^ migratory DCs (130). Even though CD8^+^ DCs were reduced in GM-CSF transgenic mice, the number of CD103^+^ DCs was increased in GM-CSF transgenic mice (135), indicating subtle differences in the two types of cDC1s differentiated at different locations. Apart from the impacts on differentiation and DC cell survival discussed above, GM-CSF has also been shown to increase the cross-presentation properties of cDC1s both *in vitro* and *in vivo* (131, 136). Functional enhancement of cDC1s by GM-CSF is also associated with an increase in CD103 expression (131, 136). However, expression of CD103 *per se* is not sufficient for acquisition of cross-presentation capacity as TGF-β increased CD103 expression but not cross-presentation of cDC1s (131). Together, GM-CSF has a broad impact not only on the processes driving DC differentiation but also affects DC effector function at the mature state. Once again, the nature and the extent of these GM-CSF induced changes may be greatly affected by the relative abundance of GM-CSF, the state of maturity and the microenvironment encountered by the cells.

## Priming End Cell Function by GM-CSF: More Than a Numbers Game?

Despite GM-CSF seeming to be redundant in the development of moDCs *in vivo* (9, 123, 124), GM-CSF is still required for function of monocytes/MØs in the induction and progression of EAE (123, 124). Here we will discuss the different aspects of impact on MØ function by GM-CSF with the caveats of certain degrees of ambiguity surrounding the definition of monocytes, moDCs and MØs *in vivo*, and the difficulty delineating the impact of GM-CSF on cell survival and function *per se* in some studies.

### Production of Cytokines and Chemokines: Priming Effect by GM-CSF

Both GM-CSF and M-CSF can generate MØs in bone marrow cultures. However, after LPS stimulation, the two factors elicit different functions. Human GM-CSF facilitates the differentiation of CD14^+^ monocytes into IL-23 producing M1 like MØs while M-CSF promotes differentiation of M2 like MØs (137). In murine systems, GM-CSF differentiated bone marrow derived MØs (GM-BMMØs) also produce more IL-12, IL-23, TNF-α, and IL-6 than M-CSF differentiated MØs (BMMØs) (138). Moreover, GM-BMMØs preferentially activated NFκB while BMMØs preferentially activated the IRF3-STAT1 axis (138, 139). From the cytokine pattern elicited, it was proposed that GM-BMMØs is “M1-like” (IL-12^hi^, IL-23^hi^, IL-10^lo^) while BMMØs is “M2-like” (IL-12^lo^, IL-23^lo^, IL-10^hi^) (138). An adoptive transfer study supported this proposal in that GM-BMMØs but not BMMØs induced a Th1 response via IL-12 production and transferred resistance to parasite infection (140). In EAE, GM-CSF responsiveness in CCR2^+^Ly6C^hi^ monocytes/moDCs was critical for disease pathogenesis, whereas GM-CSF responsiveness in cDCs or PMNs was deemed unimportant (123, 124). Moreover, GM-CSF responsiveness in CCR2^+^ cells was required for IL-1β production (124), likely from MØs but not DCs (108). Overall, these studies highlight the importance of GM-CSF in priming MØs for production of proinflammatory cytokines under TLR and NLR stimulation and provides an explanation for the adjuvant effect of GM-CSF in cancer, inflammation, and infection, even when numbers of myeloid cells are not affected.

### Antigen-Presenting Cell (APC) Function and Costimulation

An early study showed that GM-CSF enhanced APC function by increasing IL-1β production and MHC expression (141). We and others had demonstrated that GM-CSF was required for acquisition of cross-presentation capacity by cDC1s (131, 136). Bone marrow precursors cultured with GM-CSF generated CD11c^+^ cells with modest levels of CD86 and MHC II, particularly in low density cultures, whereas late addition of IL-4 dramatically increased expression of CD86 and MHC II (6). Of note, *in vivo* treatment with human GM-CSF needed co-administration of IL-4 to enhance APC function (142). These observations suggest that GM-CSF by itself has a limited capacity to up-regulate costimulatory molecules. Consequently, CD11c^+^ cells derived from GM-CSF cultures alone have a weak capacity to induce T cell proliferation compared with those derived from IL-4 supplemented cultures (6). To complicate the issue, moDCs could also suppress the APC function of cDCs (125). Overall, although GM-CSF promotes APC survival and differentiation fate, it may have limited direct effect on APC function.

### Effector Function

In the steady state, a deficiency in GM-CSF or its receptor GM-CSFR led to defective terminal differentiation of alveolar MØs, resulting in impaired surfactant catabolism and pulmonary alveolar proteinosis in both human and mice (8, 143). GM-CSF activated PU.1 to drive this differentiation pathway (144); local delivery of GM-CSF restored PU.1 and corrected the disease (144–146). In GM-CSF transgenic mice, MØs showed increased phagocytic activity and increased production of oxygen degradation products (11, 147). *In vitro*, GM-CSF primed GM-BMMØs for TLR-stimulated increased nitric oxide and lipid mediator LTB4 production but a reduction in PGE2 (148). In the absence of GM-CSF, MØs had reduced capacity for up-taking apoptotic cells (70).

## Priming End Cell Function by GM-CSF: What Determines M1 MØ or M2 MØ Deviation?

Although GM-CSF has been viewed predominantly as a pro-inflammatory cytokine and promotes differentiation of M1-like MØs that produce proinflammatory cytokines (137, 138, 149), GM-CSF has also been associated with development of M2-like MØs (47, 49). What then determined the M1-like MØ vs. M2-like MØ fate under GM-CSF stimulation? Evidence from tumor settings indicated that GM-CSF abundance was a key factor in determining cell fate. Production of high levels of GM-CSF by tumor cells led to increased M2 like MØ accumulation within the cancerous tissues, thereby inhibiting T cell response in mouse models of melanoma and pancreatic cancer (47, 49). Conversely, GM-CSF blockade reduced the development of M2 like MØs (49). It remains unclear how GM-CSF drives M2 like MØ differentiation. A study showed that GM-CSF could activate JAK2/STAT5 which in turn suppressed IRF8 transcription (150). Functionally, IRF8 could suppress M2 like MØ differentiation since IRF8 deficiency promoted M2-like MØs differentiation in tumors, while overexpression of IRF8 reduced M2-like MØ accumulation (150). Other transcription factors influenced by GM-CSF signaling in M2-like MØ activity include C/EBPbeta (151) and RORC1 (152). Interestingly, IL-3, a cytokine sharing the signaling receptor with GM-CSF, also promoted prostaglandin E2-producing M2 like MØs *in vitro* (153).

Apart from difference in cytokine production, mouse M2 MØs express high levels of characteristic markers such as Arginase 1 (Arg1), Chitinase-like 3 (Chil3, YM1), and transglutaminase 2 (Tgm2) (149, 154). These molecules had been demonstrated to mediate immunosuppression, tumor metastasis and tumor growth (155, 156). While excess GM-CSF has been associated with development of M2 like MØs (47, 49), IL-4 is also known for its potent role in shaping M2 MØ differentiation and confers many functional characteristics of M2 MØs (149). However, when IL-4 was dosed in combination with GM-CSF, M2 MØs could also differentiate into fully functional APCs (47). The coordinate action of GM-CSF and IL-4 in promoting myeloid cell fate decisions remains puzzling. We reasoned that GM-CSF and IL-4 likely instruct distinct signaling modules leading to M2 MØ differentiation. As alluded to above, GM-CSF activated STAT5 which in turn suppressed IRF8, the transcription factor suppressing M2 MØ differentiation (150). On the other hand, IL-4 promoted M2 MØ differentiation via STAT6 activation and IRF4 induction in M-CSF differentiated MØs (157, 158). To complicate the issue, GM-CSF can also induce IRF4 expression in MØs (159). IRF4 also played an important role in deciding DC vs. MØ fate, as a recent study showed that IRF4 deficiency favored MØ differentiation over DC differentiation of monocytes in the presence of IL-4 and GM-CSF (120). Overall, the signaling events emanated from GM-CSF and IL-4, leading to the differentiation of functionally distinctive DCs, M1-like MØs and M2-like MØs, have not been fully defined. In addition to IL-4, another Th2 cytokine IL-13 has been shown to suppress the production of proinflammatory cytokines (160, 161). It seems that both IL-4 and IL-13 acted in a similar fashion via STAT6 activation to modulate MØ function (162).

GM-CSF can activate PI3K and NFκB pathways promoting myeloid cell survival (19) and contributing to lung inflammation (163). However, activation of PI3K pathway can also polarize MØs (25, 75, 164). Of note, the downstream signaling molecules associated with PI3K activity, Akt1, and Akt2, have been shown to have contrasting effects in controlling MØ polarization; while the latter promotes M2 MØs, Akt1 was shown to induce M1 MØ polarization (25). An unanswered question is whether and how GM-CSF, and in particular its signaling strength, promotes differential activation of Akt1 vs. Akt2.

Finally, GM-CSF can also mediate immunosuppression indirectly via promoting Treg induction (165). GM-CSF induces the expression of milk fat globule EGF8 (MFG-E8) that promotes uptake of apoptotic cells by MØs, inducing their production of TGFβ and thereby controlling Treg development (165). Interestingly, TLR stimulation or uptake of necrotic cells was shown to downregulate MFG-E8 expression and to reduce the impact of GM-CSF on MFG-E8 expression, thus preserving the pro-inflammatory action of GM-CSF in tumor immunity (165), suggesting a pathway countering GM-CSF mediated immunosuppression.

Beyond tumors, the influence of GM-CSF on M2 like MØs extends to several inflammatory situations such as autoimmunity (67), infection (166), and transplantation (167). In general, research into the impact of GM-CSF has so far mainly focused on its property to expand myeloid cells. It still remains unclear how GM-CSF steers macrophage function to M1 MØs vs. M2 MØs. While M2 MØs may be detrimental in the context of tumor immunity, they may also be beneficial in damping autoimmunity, transplant rejection and infection-associated immunopathology and therefore it is of importance to be better define GM-CSF and its signaling components as this may avail therapeutic targets during M2 MØs development and function.

## Concluding Remarks

**Figure 2:**
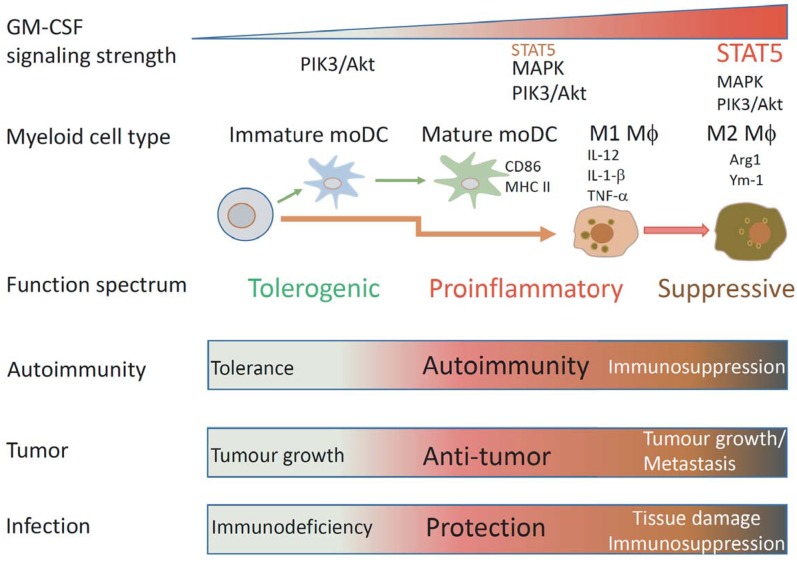
Schematic illustrating how GM-CSF signaling strength affects mononuclear myeloid cell differentiation and function. Under different GM-CSF signaling strength, different types of mononuclear myeloid cells with different functional properties are differentiated. Low GM-CSF signaling strength favors development of immature DCs, intermediated signaling strength favors development of MHCII^hi^CD86^hi^ mature DCs, high signaling strength favors development of proinflammatory M1 MØs, ultra-high signaling strength favors development of suppressive M2 MØs. According to these properties, these cells could have particular impacts on immunity to autoantigens, tumors, and infection.

GM-CSF is produced by many cells and its receptor is broadly expressed by hematopoietic cells. Engagement of GM-CSFR activates multiple signal pathways in a dose dependent manner to impact on multiple cellular processes including survival, proliferation, differentiation and function of multiple myeloid cells. Due to its promiscuous properties, GM-CSF roles in controlling pro-inflammatory or anti-inflammatory processes in healthy or diseased individuals are often complex and paradoxical. We opine that GM-CSF signaling strength likely determines biological outcome (Figure 2). At the cellular level, it drives differentiation of different cell subsets by activating different signaling modules. At the functional level, it programs antigen presentation capacity, proinflammatory function and suppressive function. Ultimately, these cellular changes will impact immunity and immunopathology in different disease settings.

In the tumor setting, relatively low to moderate doses of GM-CSF favored the immune adjuvant activity, while high doses of endogenous tumor-derived or exogenous GM-CSF could expand M2 like suppressor cells (54). GM-CSF also directly or indirectly expanded Tregs (67). For the latter, blocking GM-CSF could improve anti-tumor immunity (49). As GM-CSF mediated graft-vs.-host disease but not graft-vs.-leukemia response (77), blocking GM-CSF and receptor signaling could be also beneficial. Beyond ligand abundance, downstream signaling responsible for different cell fates should also be explored as intervention targets. Individual IRF members and Akt subunits have differential impacts on DCs, M1, and M2 MØs. SOCS family members naturally act as negative regulators as a brake on cytokine signaling. Their action can be potentially targeted to modify monocytic cell differentiation and function. Furthermore, directly targeting suppressive function of M2 MØs may also be considered. Both Arginase 1 and Chil3 are critical for arginine metabolism while arginine availability is key to an optimal T cell immune response (168). Arginase 1 inhibitor L-Norvaline and iNOS2 inhibitor L-NMMA had been found to enhance T cell proliferation (125, 169). It would be interesting to test whether selective targeting of these effector molecules of M2 MØs could enhance the beneficial anti-tumor effect of GM-CSF. In addition, IL-4 and IL-13 can dramatically change the differentiation trajectory of immune cells and their function. Therefore, their potential should also be considered when immune intervention strategies are explored.

In the autoimmune setting, anti-human GM-CSF mAb (63, 64) and anti-human GM-CSF receptor α mAb (65, 66) have also been shown to ameliorate rheumatoid arthritis in clinical trials, reinforcing the work of several decades that GM-CSF is a key proinflammatory cytokine. Yet, it remains unknown whether the tolerogenic roles of GM-CSF including expansion of Tregs (74) and induction of suppressive MØs (75) could also be harnessed. In addition, immunosuppression also occurs in chronic infections in which high levels of GM-CSF can persist (38). Perhaps, antagonism of GM-CSF in such settings could also be beneficial.

In summary, GM-CSF has pleiotropic effects on myeloid cell differentiation and function. The complexity of GM-CSF action provides a challenge but also an opportunity for tailored immune intervention. To fully capitalize on the agonistic and antagonistic effects of GM-CSF as in cancer, inflammation and infection, the differential impact of GM-CSF signaling strength on different target cells should be considered.

## Author Contributions

YZ, AL, and MC wrote the paper.

### Conflict of Interest

The authors declare that the research was conducted in the absence of any commercial or financial relationships that could be construed as a potential conflict of interest.
